# Correction: Global diabatic potential energy surfaces for the BeH_2_^+^ system and dynamics studies on the Be^+^(^2^P) + H_2_(X^1^Σ_g_^+^) → BeH^+^(X^1^Σ^+^) + H(^2^S) reaction

**DOI:** 10.1039/c8ra90057d

**Published:** 2018-07-13

**Authors:** Zijiang Yang, Jiuchuang Yuan, Shufen Wang, Maodu Chen

**Affiliations:** Key Laboratory of Materials Modification by Laser, Electron, and Ion Beams (Ministry of Education), School of Physics, Dalian University of Technology Dalian 116024 P. R. China mdchen@dlut.edu.cn

## Abstract

Correction for ‘Global diabatic potential energy surfaces for the BeH_2_^+^ system and dynamics studies on the Be^+^(^2^P) + H_2_(X^1^Σ_g_^+^) → BeH^+^(X^1^Σ^+^) + H(^2^S) reaction’ by Zijiang Yang *et al.*, *RSC Adv.*, 2018, **8**, 22823–22834.

The editorial office regrets that eqn (5)–(7) are shown incorrectly in the original manuscript. The corrected equations are shown below.

5 〈*ψ*_3_^*a*^|*P̂*|*ψ*_1_^*a*^〉 = 〈*ψ*_3_^*a*^|*P̂*|*ϕ*_1_^*d*^〉 cos *α* + 〈*ψ*_3_^*a*^|*P̂*|*ϕ*_2_^*d*^〉 sin *α*

6 〈*ψ*_3_^*a*^|*P̂*|*ψ*_2_^*a*^〉 = −〈*ψ*_3_^*a*^|*P̂*|*ϕ*_1_^*d*^〉 sin *α* + 〈*ψ*_3_^*a*^|*P̂*|*ϕ*_2_^*d*^〉 cos *α*

7
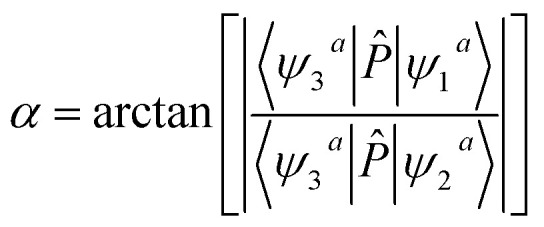


The Royal Society of Chemistry apologises for these errors and any consequent inconvenience to authors and readers.

## Supplementary Material

